# Cultural Dimensions of Workplace Violence in Healthcare: A Narrative Review

**DOI:** 10.1155/jonm/8336854

**Published:** 2025-12-22

**Authors:** Sara Morales Palomares, Flavio Gheri, Nicola Ramacciati

**Affiliations:** ^1^ Department of Pharmacy, Health and Nutritional Sciences (DFSSN), University of Calabria, Rende, Italy, unical.it; ^2^ Department of Biomedicine and Prevention, University of Rome “Tor Vergata”, Rome, Italy, uniroma2.it

**Keywords:** cultural factors, healthcare workers, workplace violence

## Abstract

**Introduction:**

Workplace violence (WPV) against healthcare workers is a global concern, affecting safety, well‐being, and job satisfaction. Cultural factors significantly influence aggression dynamics, making their understanding crucial for targeted prevention strategies.

**Aim:**

This study aims to identify and analyze the cultural factors contributing to WPV and to explore how these elements influence both aggression dynamics and the effectiveness of strategies for its prevention and mitigation.

**Methods:**

A narrative review was conducted in accordance with PRISMA guidelines. Literature searches in PubMed, CINAHL, and Scopus identified studies on cultural dimensions of WPV in healthcare. A thematic analysis synthesized findings.

**Results:**

Eight studies were included in the review. The analysis identified four primary cultural dimensions contributing to WPV: (1) Ethnic and linguistic disparities: Healthcare workers from minority backgrounds face higher risks due to language barriers and cultural biases, leading to miscommunication and increased aggression. (2) Female healthcare professionals, particularly in patriarchal or hierarchical workplaces, are disproportionately affected by WPV due to structural power imbalances and gender stereotypes. (3) Sociocultural perceptions of healthcare workers: Patients’ expectations of “unconditional care” can heighten frustration and aggression when unmet, particularly in underfunded or resource‐limited healthcare settings. (4) Cultural normalization and underreporting: In many cultures, WPV is perceived as an inherent part of the profession, discouraging formal reporting and institutional responses.

**Conclusion:**

Cultural factors shape WPV prevalence, reporting, and management. Addressing these factors through culturally competent interventions, language accessibility, gender‐sensitive policies, and institutional reforms is essential for safer workplaces. Future research should standardize WPV prevention strategies integrating cultural awareness and cross‐cultural communication training.

**Implications for Nursing Management:**

Nursing management plays a key role in WPV prevention through culturally competent strategies, gender‐sensitive policies, and systemic reforms. Implementing cultural competence training, language accessibility, and zero‐tolerance policies can enhance safety. Strengthening reporting mechanisms and advocating for adequate staffing are crucial steps in mitigating WPV and fostering a safer healthcare environment.

## 1. Introduction

Workplace violence (WPV) is a critical global public health issue that poses significant threats to healthcare professionals, compromising their physical safety, mental well‐being, and job satisfaction [1, 2]. Defined by the World Health Organization (WHO) as incidents where workers are abused, threatened, or assaulted in work‐related circumstances, WPV can manifest in both physical and psychological forms [3]. This pervasive issue has drawn considerable international attention, particularly in contexts where the working conditions of healthcare workers are increasingly strained [4].

Healthcare professionals across various medical settings frequently encounter aggression from patients, their families, or even colleagues. This creates a hostile work environment that not only impacts the well‐being of healthcare workers but also undermines the quality of care delivered to patients [2, 5, 6]. According to a recent WHO report, approximately 8% and 38% of healthcare workers worldwide have experienced some form of violence during their professional careers [7]. Despite its alarming prevalence, WPV remains significantly underreported worldwide. This chronic underreporting is often driven by cultural stigma, the normalization of violence, and systemic barriers that discourage healthcare professionals from disclosing incidents [8]. In some cultures, violence is even perceived as an inevitable part of healthcare work, a belief that perpetuates the cycle of aggression and obstructs efforts to develop effective prevention strategies [9]. The consequences of WPV extend beyond immediate physical harm, as healthcare workers frequently experience psychological impacts such as burnout, post‐traumatic stress disorder, and decreased job satisfaction [10, 11]. These outcomes highlight the urgent need for comprehensive interventions aimed at fostering safer and more supportive work environments [12].

While extensive research has examined WPV through organizational and individual lenses, cultural dimensions remain underexplored despite their significant influence on the dynamics of aggression. Societal norms, linguistic barriers, and hierarchical workplace expectations are critical in shaping the dynamics of aggression in healthcare settings. For instance, cultural stereotypes and the perception of healthcare workers as providers of unconditional care can heighten tensions, leading to violence when these expectations are unmet [13]. Healthcare professionals from minority backgrounds are particularly vulnerable to WPV due to biases, cultural misunderstandings, and communication challenges in multicultural work environments [14]. Furthermore, in some cultures, violence against healthcare workers is perceived as an inevitable part of the profession, leading to significant underreporting and a lack of adequate responses [5, 15].

Although WPV is increasingly recognized as a multifaceted problem [16], the cultural dimensions driving such aggression remain insufficiently addressed in both research and practice. Understanding these factors is essential for developing effective interventions that mitigate violence, foster inclusivity, and promote mutual respect in healthcare environments. This narrative review examines the intersection of cultural factors and WPV in healthcare. By analyzing the cultural elements influencing aggression and synthesizing findings from existing literature, this study provides a foundation for developing culturally informed interventions. These insights are important to contribute to the creation of culturally informed strategies to reduce WPV, improve workplace safety, and enhance cross‐cultural understanding within healthcare settings.

### 1.1. Aim

This study aims to identify and analyze the cultural factors contributing to WPV against healthcare workers and to examine the strategies that have been proposed or implemented to address these cultural determinants, with the goal of informing targeted interventions to prevent or mitigate WPV.

## 2. Materials and Methods

### 2.1. Study Design

A narrative literature review was conducted to examine the cultural dimensions of WPV in healthcare. This review followed Sukhera’s narrative review methodology [17] and incorporated selected elements of the Preferred Reporting Items for Systematic Reviews and Meta‐Analyses (PRISMA) guidelines [18] solely to enhance transparency and reporting clarity. The study sought to answer the following research question: What are the key cultural factors influencing WPV against healthcare workers, and what strategies have been proposed or implemented to address these cultural determinants and reduce the risk of WPV?

### 2.2. Definition of Research Question

The research question was developed using the PICO framework [19] to ensure a structured and comprehensive approach: P (Population): Healthcare workers in diverse cultural and professional settings. I (Intervention): Strategies or factors addressing cultural dimensions in WPV. C (Comparison): Healthcare environments with varying cultural norms or interventions addressing cultural aspects versus no interventions. O (Outcomes): Identification of key cultural determinants of WPV, understanding their effects on aggression dynamics, and evaluating strategies aimed at reducing or preventing WPV in multicultural healthcare contexts.

### 2.3. Eligibility Criteria

The inclusion and exclusion criteria for this narrative review were meticulously designed to ensure the selection of studies directly relevant to the research question while maintaining methodological rigor. These criteria were iteratively refined during database searches to align with the review’s objectives. Inclusion criteria were as follows: peer‐reviewed, full‐text articles in English; conducted in healthcare settings; participants were healthcare workers (any profession). Studies had to (i) explicitly examine cultural determinants of WPV (e.g., ethnicity/language, gender norms, hierarchy/roles, social norms) or (ii) evaluate strategies or policies targeting those cultural determinants. Eligible designs included empirical studies (quantitative, qualitative, or mixed‐methods) and evidence syntheses with transparent methods. No date limits were applied.

Exclusion criteria were as follows: non–peer‐reviewed items (e.g., guidelines, editorials, commentaries); abstract‐only records; studies conducted in non‐healthcare settings or involving non‐healthcare populations; and studies on WPV that did not include an explicit cultural focus as a primary aim.

### 2.4. Search Strategy

The search strategy for this narrative review was conducted using the databases PubMed, Cumulative Index to Nursing and Allied Health Literature (CINAHL), and Scopus to ensure comprehensive coverage of relevant literature on the cultural dimensions of WPV in healthcare. The development of search strings involved the use of controlled vocabulary terms, such as MeSH terms, alongside additional keywords and Boolean operators. These search strings were tailored to the specific characteristics of each database consulted. Keywords such as “workplace violence,” “healthcare workers,” and “cultural factors,” along with their synonyms and related terms, were combined using the Boolean operators AND and OR to align with the study’s eligibility criteria. Following the initial search to identify the total number of records, the article screening process was carried out independently by two researchers. Any disagreements regarding study inclusion were resolved through discussion with a third researcher, ensuring consistency and methodological rigor. Rayyan software (available at https://www.rayyan.ai) was utilized to organize and systematically manage the bibliographic records. Detailed search terms and the full search strategy are provided in Supporting Table S1.

### 2.5. Data Synthesis

To systematically address the research questions and objectives of this narrative review on WPV in healthcare, a structured data extraction process was implemented. A data extraction table was developed, including key elements such as author information, year of publication, country of study, study design, aim of the study, cultural factors contributing to WPV, and strategies for addressing cultural factors in WPV (Table 1). This systematic approach facilitated the synthesis of relevant findings, offering a comprehensive understanding of the cultural dimensions of WPV in healthcare.

**Table 1:** Characteristics of the included studies.

Authors and year	Country	Study design	Aim of the study	Cultural factors contributing to WPV	Strategies for addressing cultural factors in WPV
Pasquini, 2023 [20]	Italy	Ethnographic‐qualitative study	Explore WPV experiences in emergency departments	1. Structural violence (underfunding, overcrowding). 2. Cultural perceptions of nurses (unrealistic expectations). 3. WPV normalization.	1. Structural reforms (staffing, funding) 2. Awareness campaigns. 3. Institutional accountability.

Bagnasco et al. 2024 [21]	Italy	Cross‐sectional study	Identify risk and protective factors of WPV against nurses	1. Patient cultural background as WPV predictor. 2. WPV seen as inevitable. 3. Underreporting due to cultural acceptance.	1. Cultural competence training. 2. Policies rejecting WPV normalization. 3. Enhanced reporting mechanisms.

Sabri et al. 2015 [22]	USA	Cross‐sectional study	Examine racial/ethnic differences in WPV exposure	1. Racial/ethnic disparities in WPV. 2. Underutilization of WPV resources among minorities. 3. Cultural values influencing WPV reporting.	1. Culturally tailored training. 2. Mandatory workplace education. 3. Addressing racial/ethnic vulnerabilities in WPV interventions.

Ayaz et al. 2024 [23]	Canada	Scoping review	Map WPV prevalence with gender and cultural focus	1. Gender norms: Women in lower‐status positions face higher WPV. 2. Underreporting due to cultural stigma. 3. Gender disparity in leadership roles.	1. Zero‐tolerance policies. 2. Culturally sensitive training. 3. Transparent reporting mechanisms.

Lisak et al. 2021 [24]	Israel	Cross‐sectional study	Influence of cultural factors on patient satisfaction and aggression in ED	1. Cultural affiliation impacts satisfaction and aggression 2. Language accessibility issues.	1. Language‐accessible medical info. 2. Cultural competency training. 3. Inclusive hospital policies.

Jia et al. 2020 [25]	China	Cross‐sectional study	WPV in multiethnic China, with focus on healthcare professionals’ responses	1. Ethnic minority status linked to higher WPV risk. 2. Cultural reluctance to report WPV.	1. Increased security measures. 2. Targeted cultural training. 3. Simplified WPV reporting procedures.

Shohani and Tavan, 2024 [26]	Iran	Systematic review and meta‐analysis	Investigate cultural violence against nurses	1. Ethnic and racial discrimination in WPV. 2. Higher risk in emergency/psychiatric units.	1. Cultural competence training. 2. Improved working conditions. 3. Reporting systems.

Büyükbayram and Okçay, 2013 [27]	Turkey	Review article	Identify sociocultural factors influencing violence against healthcare workers	1. Cultural acceptance of violence. 2. Gender dynamics and violence risks. 3. Media portrayals of healthcare workers.	1. Legal reforms and public awareness campaigns. 2. Workplace training and conflict resolution. 3. Media regulation.

## 3. Results

### 3.1. Screening

**Figure 1:**
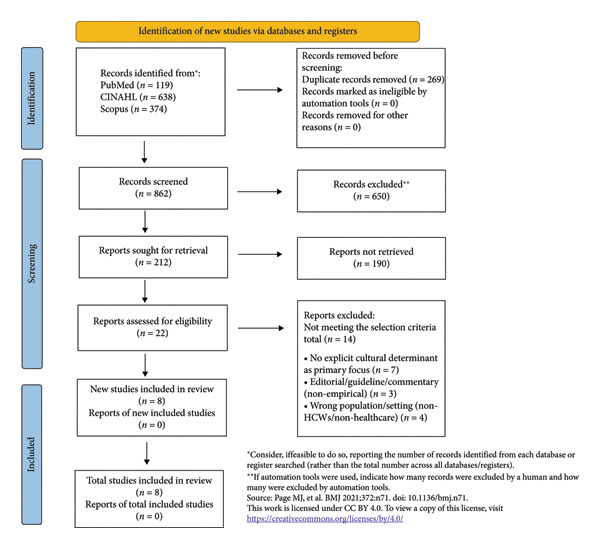
PRISMA ScR flowchart.

From a total of 1131 identified records through searches in various databases: PubMed‐Medline (*n* = 119), CINAHL (*n* = 638), and Scopus (*n* = 374), 269 duplicates were removed. Subsequently, the titles and abstracts of 862 unique records were screened. Based on title relevance, 212 articles were selected for further evaluation through abstract review. During this phase, 190 articles were excluded as irrelevant. Subsequently, 22 full‐text articles were assessed for eligibility. Of these, 14 studies were excluded for the following reasons: no explicit cultural determinant as a primary focus (*n* = 7); editorial/guideline/commentary (nonempirical) (*n* = 3); wrong population/setting (non‐HCWs/non‐healthcare) (*n* = 4). After the screening process, a total of eight studies were included in this narrative review (Figure 1).

### 3.2. Characteristics of the Studies Included

The review included a total of eight studies published between 2013 and 2024. Geographically, the studies were conducted in various countries: Italy (*n* = 2), the United States (*n* = 1), Canada (*n* = 1), Israel (*n* = 1), China (*n* = 1), Iran (*n* = 1), and Turkey (*n* = 1). The research designs varied across the studies, comprising four cross‐sectional studies [21, 22, 24, 25], three review articles [23, 26, 27], and one ethnographic qualitative study [20]. The review identified multiple cultural factors contributing to WPV in healthcare, which influence both the prevalence and management of aggression toward healthcare workers. Thematic analysis revealed four main cultural dimensions associated with WPV: ethnic and linguistic disparities [22, 24–26], gender and hierarchical norms [23, 27], sociocultural perceptions of healthcare workers [20, 21, 24, 27], and underreporting due to cultural normalization [20, 22, 25, 27]. Additionally, strategies for addressing WPV in multicultural healthcare settings were explored across multiple studies [20–27]. Characteristics of the 8 included studies in the review are provided in Table 1.

### 3.3. Ethnic and Linguistic Disparities in WPV

Research highlights that ethnic and linguistic disparities in healthcare settings significantly contribute to an increased risk of WPV. Healthcare workers from minority backgrounds are disproportionately affected by verbal aggression, bullying, and physical assaults compared to their majority‐group colleagues [22, 25]. A cross‐sectional study conducted by Jia et al. [25] in a multiethnic region of Southwest China investigated how ethnic and linguistic disparities contribute to WPV. The findings demonstrated that minority healthcare workers were at a significantly higher risk of experiencing psychological violence, particularly verbal aggression and threats from patients and their families. Similarly, a study by Sabri et al. [22] in the United States examined racial and ethnic differences in WPV victimization and found that Black and Asian nurses were less likely to be aware of WPV prevention resources compared to their White colleagues. The study also revealed that minority healthcare workers were less inclined to report violent incidents due to fear of retaliation and perceived institutional inaction, indicating that systemic discrimination and cultural misunderstandings increase their vulnerability to WPV.

Linguistic barriers have been identified as a significant factor contributing to WPV, particularly in multilingual healthcare settings. Patients with limited proficiency in the dominant language often struggle to communicate their needs effectively, leading to frustration and, in some cases, aggression [24–26]. A study by Lisak et al. [24] conducted in Israel found that patients from minority cultural groups who lacked access to healthcare information in their native language reported higher levels of dissatisfaction, which in turn increased their aggressive behavior toward medical staff. Similarly, Jia et al. [25] reported that healthcare workers in China who faced challenges communicating in Mandarin were at greater risk of experiencing WPV, particularly from patients who perceived them as less competent due to language differences. Building on these findings, the recent systematic review by Shohani & Tavan [26] further emphasized that healthcare professionals working in multilingual settings, particularly in emergency departments, were more likely to experience verbal aggression from patients who struggled to express their needs. The review highlighted that language barriers, especially in high‐stress environments, exacerbate workplace tensions and increase the likelihood of violent interactions.

### 3.4. Gender and Hierarchical Norms in WPV

The reviewed literature underscores that gender‐based WPV is a pervasive issue in healthcare settings, disproportionately affecting female healthcare workers. The studies indicate that deeply ingrained cultural norms, particularly in patriarchal societies, contribute to the normalization of verbal abuse and sexual harassment against women in healthcare professions [23, 27]. A study by Büyükbayram and Okçay [27] in Turkey examined the impact of cultural norms on WPV and found that female healthcare workers frequently experience verbal abuse and sexual harassment, particularly in societies where patriarchal structures position them in subordinate roles within the healthcare hierarchy. The study also highlighted that women are often reluctant to formally report violence due to fear of retaliation and a lack of institutional support. More recently, a scoping review by Ayaz et al. [23] reinforced these findings, demonstrating that structural and sociocultural factors contribute to gender disparities in WPV. The review emphasized that women in healthcare are overrepresented in lower paid positions, have limited access to leadership roles, and are subjected to cultural stereotypes that normalize abusive behaviors. The study also found that sexual harassment is predominantly perpetrated by male superiors or patients, further reinforcing existing power imbalances.

Beyond gender‐based disparities, workplace power dynamics play a crucial role in WPV. Büyükbayram and Okçay [27] noted that ineffective institutional responses leave affected workers without adequate support, highlighting the urgent need for targeted strategies to address gender‐based violence in healthcare settings. Similarly, Ayaz et al. [23] observed that men in leadership roles frequently exert influence in ways that marginalize female colleagues, either through direct actions or by sustaining a workplace culture that tolerates gender‐based discrimination.

### 3.5. Sociocultural Perceptions of Healthcare Workers and WPV

The included studies suggest that sociocultural perceptions of healthcare professionals play a crucial role in influencing both the prevalence and nature of WPV. In many cultural contexts, healthcare workers are viewed as providers of “unconditional care,” leading to unrealistic expectations from patients and their families. When these expectations clash with systemic constraints such as long wait times, understaffing, and limited access to treatment, frustration often escalates into aggression against medical staff [20, 21, 24, 27]. As previously noted, Büyükbayram and Okçay [27] examined how sociocultural factors contribute to WPV in Turkey. Their study further highlighted that deep‐rooted societal norms, including hierarchical structures within healthcare and patriarchal attitudes, influence how violence is perceived and tolerated. Nurses and other frontline healthcare workers, particularly women, were found to be at higher risk of verbal abuse and physical aggression, as their roles are often viewed as subordinate. Additionally, substance use, mental health disorders, and cultural perceptions of authority were identified as key predictors of WPV in healthcare settings. Similarly, Lisak et al. [24] investigated how cultural perceptions influence violence in Israeli emergency departments. Their study found that patients from minority backgrounds who perceived inequitable treatment were more likely to exhibit aggressive behavior toward healthcare staff. In particular, the lack of access to healthcare information in one’s native language was associated with higher levels of dissatisfaction, intensifying tensions between patients and providers. Expanding on these findings, Pasquini [20] conducted an ethnographic study on WPV in Italian emergency departments. The research suggested that healthcare workers often perceive violence as an inevitable part of the job rather than a personal attack, reflecting broader structural challenges in the healthcare system. Chronic underfunding, overcrowding, and resource scarcity were identified as key contributors to patient frustration and aggression. Moreover, medical professionals in gatekeeping roles, those responsible for making treatment decisions, were more frequently targeted by violent incidents due to their perceived control over access to care. Providing further evidence, Bagnasco et al. [21] found that authoritarian attitudes toward medical professionals, particularly nurses, contribute to the normalization of verbal and physical aggression. In patriarchal societies, nurses are often viewed as subordinate figures within the healthcare hierarchy, making them more vulnerable to violence. The study also noted that WPV is frequently dismissed as an occupational hazard, discouraging formal reporting and institutional intervention.

### 3.6. Cultural Normalization and Underreporting of WPV

Findings from the included studies indicate that underreporting of WPV in healthcare settings remains a persistent issue, particularly in cultural contexts where aggression is normalized. This phenomenon arises from a combination of sociocultural attitudes, professional expectations, and institutional shortcomings that discourage healthcare workers from formally reporting violent incidents [20, 22, 25, 27]. Büyükbayram and Okçay [27] found that violence against healthcare professionals is often perceived as an unavoidable occupational hazard, especially in high‐stress environments such as emergency departments and psychiatric units. The normalization of violence discourages reporting, as many healthcare workers consider such incidents part of their professional reality rather than reportable offenses. Additionally, broader societal attitudes reinforce the acceptance of aggression, particularly when linked to patient frustrations over long wait times, treatment denial, or perceived inequities in healthcare access. Similarly, Pasquini [20] observed that healthcare professionals in Italian emergency services often interpret WPV as a structural issue rather than an act of individual aggression. Understaffing, resource limitations, and systemic inefficiencies are frequently cited as primary contributors to WPV, leading many workers to normalize violence and refrain from filing official complaints.

In addition to cultural perceptions of violence, a major deterrent to reporting is the widespread belief that institutional responses are ineffective. Many healthcare professionals feel that filing a report will not lead to meaningful action or, in some cases, may even result in negative repercussions [22, 25]. Hospital administrators may be reluctant to address WPV due to the absence of effective policies, lack of enforcement mechanisms, or concerns over institutional reputation. This fosters a culture of self‐silencing, in which healthcare workers fear that reporting violence may jeopardize their professional standing, lead to reduced work shifts, or make them appear less competent. This issue is particularly pronounced among minority healthcare workers, who often face additional barriers to reporting WPV. Sabri et al. [22] found that racial and ethnic minority healthcare professionals are less likely to report incidents of violence due to fears of discrimination, lack of managerial support, and concerns about retaliation. These findings underscore broader power imbalances within healthcare institutions, where marginalized workers may feel vulnerable not only to patient aggression but also to institutional neglect.

The consequences of underreporting WPV are profound. A cross‐sectional study by Jia et al. [25] found that most WPV incidents involve verbal abuse, intimidation, and threats, yet these cases frequently go undocumented because they do not meet the threshold for formal intervention. Even physical assaults often remain unreported, as healthcare professionals may perceive them as routine occurrences rather than exceptional incidents requiring intervention. The study emphasized that underreporting perpetuates a cycle in which healthcare workers refrain from reporting WPV due to skepticism about institutional responses, institutions underestimate the prevalence of violence, perpetrators face no consequences, and the overall mental and emotional toll on healthcare workers increases. This ultimately contributes to burnout, job dissatisfaction, and high turnover rates.

### 3.7. Strategies for Addressing WPV in Multicultural Healthcare Settings

The reviewed studies emphasize that addressing WPV in multicultural healthcare settings requires a multifaceted approach that integrates institutional policies, cultural competence training, and systemic reforms. Research highlights the importance of tailoring strategies to the specific cultural, linguistic, and structural factors that contribute to WPV, rather than relying solely on individual healthcare workers to manage violent incidents. Several studies have proposed interventions aimed at mitigating violence by improving communication, strengthening institutional responses, and fostering a more inclusive and supportive work environment.

According to several studies, one widely endorsed strategy is cultural competency training, which enhances healthcare workers’ ability to navigate diverse patient interactions and prevent conflicts before they escalate. Research indicates that training programs focusing on de‐escalation techniques, recognition of cultural variations in communication, and increased awareness of patient expectations can significantly reduce aggression [24, 26]. In particular, multilingual education and interpretation services have been identified as essential tools for reducing misunderstandings, as language barriers have been shown to exacerbate patient dissatisfaction and increase the risk of aggression in healthcare settings [24, 25]. By equipping medical staff with cross‐cultural communication skills and providing access to real‐time interpretation services, healthcare institutions can foster more positive patient–provider relationships and minimize conflict.

Given the prevalence of gender‐based WPV, particularly against female healthcare workers, studies emphasize the need for institutional interventions that promote gender equality and protect women in healthcare professions. Research highlights that gender‐sensitive policies, such as zero‐tolerance measures for harassment and abuse, are essential in preventing violence and creating safer work environments [23, 27]. Additionally, training programs specifically designed for female healthcare workers focusing on self‐defense and conflict resolution have been identified as effective tools for increasing confidence and preparedness in high‐risk situations [23].

Another key intervention identified in the included studies is the implementation of clear antiviolence policies that explicitly account for cultural influences on WPV. Institutional guidelines should clearly define unacceptable behaviors, establish transparent reporting procedures, and protect healthcare workers from retaliation when incidents are reported [22, 25]. Research suggests that workplace policies are most effective when they incorporate mandatory reporting mechanisms and ensure that staff are trained to recognize and address violence, particularly in culturally diverse environments [21]. Policies should also consider sociocultural factors that influence patient aggression, such as frustration due to systemic inefficiencies, long wait times, or perceived disparities in care access [20]. Addressing these structural factors is crucial for reducing patient dissatisfaction and mitigating WPV. To combat the underreporting of WPV, strengthening reporting mechanisms and ensuring that institutional responses are effective and protective rather than punitive is critical. Research has shown that many healthcare workers, particularly those from minority backgrounds, refrain from reporting violent incidents due to fears of retaliation or perceived lack of institutional support [22, 25]. Implementing anonymous reporting systems and guaranteeing protections for those who report violence can help break the cycle of silence and encourage staff to document incidents more frequently [21]. Moreover, regular monitoring of WPV data allows institutions to identify high‐risk areas and implement targeted interventions, such as increasing security presence in emergency departments or adjusting staffing models to reduce burnout and stress [20].

**Figure 2:**
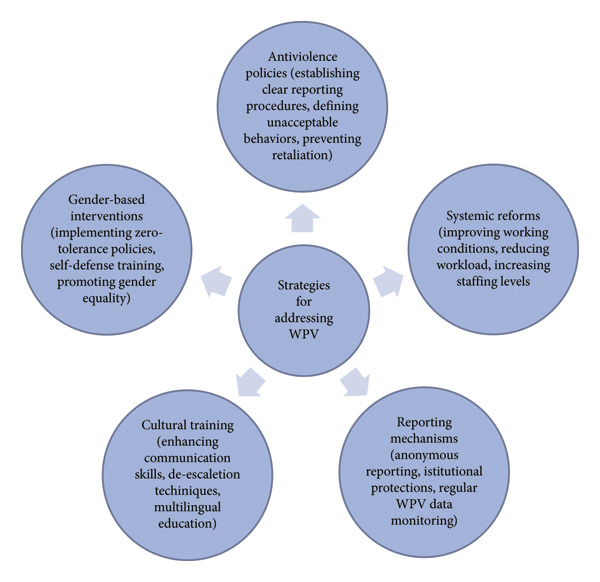
Strategies for addressing WPV.

A final crucial strategy identified in the included studies is the integration of systemic reforms that address workplace conditions contributing to WPV. Studies emphasize that WPV should not be viewed as an isolated issue but as part of a broader structural problem tied to underfunding, overcrowding, and inequitable resource distribution. Improving working conditions, reducing excessive workloads, and ensuring adequate staffing levels are essential steps in minimizing stress‐induced violence. Additionally, fostering a culture of institutional accountability, where violence prevention is prioritized and actively enforced, can help shift the perception that WPV is an inevitable aspect of healthcare work [20, 21]. Figure 2 summarizes the key strategies for addressing WPV in healthcare settings. Figure 2 provides an overview of the strategies for addressing WPV in healthcare settings.

## 4. Discussion

This narrative review analyzed the cultural factors influencing WPV in healthcare settings, highlighting how ethnic and linguistic disparities, gender norms, and sociocultural perceptions of healthcare professionals shape aggression dynamics. The findings confirm that WPV is not an isolated phenomenon but is deeply rooted in the sociocultural and organizational structures of healthcare institutions.

These cultural dimensions can be further interpreted through Hofstede’s framework. In particular, high power distance cultures, where hierarchical relationships are strongly emphasized, may amplify aggression dynamics by discouraging open communication and reinforcing authority gaps between patients and healthcare workers. Similarly, in collectivist contexts, perceived threats to group harmony can intensify conflicts with “outsider” staff, especially those from linguistic or ethnic minorities [28].

One of the most significant findings of this review is the role of ethnic and linguistic barriers in increasing the risk of WPV against healthcare workers. Research has emphasized how communication difficulties can lead to frustration among patients, particularly in emergency settings, escalating into aggression [24–26].

Recent evidence from Aca et al., [29] further supports the role of sociocultural factors in shaping WPV in healthcare. The study highlights how patient–provider interactions are deeply influenced by cultural expectations, with misaligned perceptions of authority and care responsibilities escalating conflict. Additionally, the research emphasizes the impact of organizational policies in either mitigating or exacerbating WPV, particularly when institutional responses fail to acknowledge the cultural determinants of aggression. These findings align with previous studies suggesting that effective prevention strategies should integrate culturally competent training and systemic policy reforms to foster safer working environments. A meta‐analysis found that the overall prevalence of WPV against healthcare professionals was 62.4%, with verbal abuse accounting for 61.2% of incidents [2]. Additionally, language and cultural differences have been identified as key risk factors for WPV, as they contribute to miscommunication and heightened tensions. The lack of interpreters and adequate linguistic resources further exacerbates this issue, increasing the likelihood of aggression in healthcare settings [2]. Providing cultural competence training for healthcare staff and implementing language mediation services could be key strategies in reducing WPV in multicultural healthcare environments [30]. Cultural competence training has been shown to improve the knowledge, attitudes, and skills of health professionals, thereby enhancing patient–provider interactions [31].

Additionally, the analysis of gender and hierarchical norms highlights how female healthcare professionals are particularly vulnerable to WPV, especially in patriarchal societies or hierarchical workplaces where power imbalances are more pronounced [23, 27]. From an intersectionality perspective, gender does not act in isolation but intersects with other social identities (e.g., ethnicity, age, professional role) to shape unique vulnerabilities to WPV. This lens helps explain why women from minority groups or in lower status positions face disproportionately high risks of aggression [32, 33].

Research has further emphasized these disparities, indicating that female nurses face a greater risk of aggression compared to their male counterparts [34]. A recent systematic review found that violence in both the workplace and broader society is rooted in gendered socioeconomic, cultural, and institutional factors, underscoring the need for gender‐sensitive interventions [35]. The perception of female healthcare workers as subordinate figures rather than clinical decision‐makers contributes to the normalization of violence against them. This observation aligns with previous studies demonstrating that gender discrimination and limited female representation in leadership roles amplify their exposure to WPV [36–38].

Furthermore, a qualitative study conducted in Jordan identified multiple triggers of WPV in emergency departments, emphasizing the role of aggressors’ misconceptions, social customs, and organizational factors [39]. The study found that patients and their relatives often misunderstand triage protocols, leading to frustration and conflicts with healthcare workers. Additionally, entrenched social norms such as nepotism and the use of informal reconciliation practices (“cup of coffee”) contribute to the normalization of WPV and limit legal consequences for perpetrators. From an organizational perspective, inadequate staffing and ineffective security measures were identified as key factors exacerbating WPV. This aligns with prior research emphasizing the need for systemic interventions, including stronger institutional policies and improved security personnel training, to mitigate WPV in emergency settings.

Another critical issue is the normalization of WPV and the inadequacy of reporting mechanisms. Fear of retaliation, lack of trust in institutional responses, and the perception of WPV as an inevitable part of healthcare work contribute to significant underreporting [20, 22, 25, 27]. The persistent normalization and underreporting of WPV can be interpreted through the framework of structural violence. This concept emphasizes how institutional norms, policies, and resource distributions can perpetuate harm by embedding inequities into routine practices [40, 41]. The perception of violence as “part of the job” reflects a structural acceptance of harm that discourages reporting and limits systemic responses, illustrating how organizational structures can indirectly sustain conditions of violence over time.

Strengthening reporting systems by incorporating anonymous reporting options and protective measures for those who disclose incidents could encourage healthcare workers to report violence and ensure timely institutional responses [42, 43].

Many current WPV mitigation strategies focus primarily on individual interventions rather than addressing systemic reforms. However, this review underscores the importance of adopting a holistic, multidimensional approach that addresses structural, educational, and institutional factors [20, 21, 44]. Similarly, recent qualitative research highlights that WPV is embedded within broader social and institutional structures, requiring systemic organizational changes rather than solely relying on individual‐level interventions [45]. Furthermore, research suggests that while WPV is influenced by cultural factors, there are significant similarities in the experiences of emergency nurses worldwide. This suggests that effective prevention strategies should balance culturally tailored interventions with universally applicable measures that address common WPV challenges across healthcare settings [46]. A comprehensive strategy should integrate cultural competence training, language accessibility measures, and robust institutional policies to foster a safer and more inclusive healthcare environment [20–27]. A key intervention involves integrating cultural competence training into healthcare education and professional development programs. Providing staff with cross‐cultural communication skills and de‐escalation techniques can help prevent conflicts arising from linguistic and cultural misunderstandings. Consistent with this perspective, two reviews have highlighted that enhancing healthcare professionals’ cultural awareness and patient‐centered communication strategies can improve patient–provider interactions and reduce potential conflicts [30, 31]. Similarly, the Treatment Improvement Protocol (TIP) from the Substance Abuse and Mental Health Services Administration (SAMHSA) emphasizes that culturally competent healthcare services enhance communication and patient satisfaction, thereby mitigating frustration that may escalate into aggression [47]. Beyond training, language accessibility measures play a crucial role in reducing misunderstandings that contribute to patient frustration and aggression. The availability of professional interpreters, multilingual healthcare materials, and real‐time translation technologies significantly improves patient–provider communication [48]. A study by the Occupational Safety and Health Administration (OSHA) found that language barriers were a contributing factor in many reported WPV cases, emphasizing the need for effective language support policies in healthcare settings [49]. Moreover, implementing zero‐tolerance policies is crucial for fostering a safe work environment. Establishing clear protocols for incident reporting, victim protection, and institutional responses enhances accountability and encourages timely reporting of violent incidents [50]. Strengthening workplace reporting mechanisms, including anonymous and protected reporting channels, is crucial in addressing underreporting, a persistent challenge in WPV cases [42, 43]. Studies indicate that fear of retaliation, lack of trust in the system, and the normalization of WPV prevent many healthcare workers from reporting incidents [30]. Additionally, research indicates that exposure to WPV significantly affects nurses’ psychological and physical well‐being, leading to increased stress, burnout, and even chronic physical health conditions [45]. These adverse effects can contribute to staff turnover, absenteeism, and further disruptions in healthcare service delivery. Finally, addressing gender disparities in WPV is also critical [51]. In line with this review, research suggests that integrating gender‐sensitive WPV prevention strategies can reduce the prevalence of gender‐based violence in healthcare settings [35]. Policies should include targeted measures to protect female healthcare workers from violence, harassment, and discrimination, while also promoting gender equity in leadership roles to challenge hierarchical structures that perpetuate WPV. By adopting a comprehensive and evidence‐based approach, healthcare institutions can develop sustainable interventions that not only mitigate WPV but also promote a culture of respect and safety for healthcare professionals.

Across the countries represented (Italy, USA, Canada, Israel, China, Iran, and Turkey), several cross‐cutting cultural mechanisms emerged across contexts. Ethnic and linguistic disparities, hierarchical power structures, and gendered expectations consistently increased the risk of WPV, suggesting common drivers of aggression in healthcare. At the same time, context‐specific patterns were evident. For example, studies from more collectivist or patriarchal settings (e.g., Iran, Turkey, parts of China) emphasized deference to authority and gender hierarchies that may normalize violence and suppress reporting. In contrast, studies from more individualist contexts (e.g., Canada, USA) highlighted legal accountability and patient autonomy yet still reported underreporting and gender disparities. These findings suggest that while core cultural determinants appear broadly applicable, their manifestations are shaped by local sociocultural and institutional contexts. Consequently, WPV prevention strategies should combine universal components (e.g., zero‐tolerance policies, reporting protections, cultural competence training) with context‐tailored interventions that address the specific cultural norms of each healthcare system. Such an approach would enhance the transferability and effectiveness of WPV mitigation efforts in increasingly multicultural healthcare environments.

### 4.1. Study Limitation

This review has several limitations that should be considered when interpreting its findings. The heterogeneity of the included studies, with diverse methodologies and geographic contexts, may limit the generalizability of the conclusions. The lack of standardized in measurement criteria across studies may have influenced the comparability of results. Most included studies were conducted in upper‐middle and high‐income countries (Italy, USA, Canada, Israel, China, Iran, Turkey); no studies from low‐income countries and none from sub‐Saharan Africa or Latin America were identified. Because cultural norms shaping WPV (e.g., gender relations, hierarchical structures, reporting practices) are context‐bound, the transferability of our findings to these underrepresented regions is uncertain. In addition, our inclusion of English‐language, peer‐reviewed full texts only may have excluded relevant evidence from francophone, lusophone, or Spanish‐speaking contexts. Another limitation is the absence of longitudinal data, as most of the included studies are cross‐sectional, offering limited insights into the causal relationship between cultural factors and WPV over time. Future research should prioritize primary studies in low‐income and underrepresented regions, employ culturally grounded frameworks, and use locally validated instruments and reporting standards. Expanding research efforts to explore WPV in diverse healthcare settings would help create a more globally representative perspective on this critical issue.

### 4.2. Implications for Nursing Management

Nursing management plays a pivotal role in mitigating WPV through culturally informed and systemic strategies. Given the significant impact of WPV on healthcare professionals’ well‐being and patient care quality, nursing leaders must prioritize comprehensive interventions tailored to the cultural and structural factors contributing to violence. Cultural competence training is essential for equipping healthcare workers with the necessary skills to navigate diverse patient interactions, reduce miscommunication, and effectively de‐escalate conflicts. Implementing language accessibility measures, such as professional interpreters and multilingual resources, can reduce frustration and aggression stemming from communication barriers. Gender‐sensitive policies are critical to addressing the disproportionate impact of WPV on female healthcare professionals. Nursing management should enforce zero‐tolerance policies against harassment and violence while actively promoting gender equity in leadership roles to address systemic power imbalances. Addressing underreporting requires establishing anonymous and protected reporting systems to encourage staff to document WPV incidents without fear of retaliation. Transparent institutional responses and regular monitoring of WPV data will allow for targeted interventions and policy adjustments. Finally, systemic reforms must be enacted to mitigate the stressors contributing to WPV. Nursing leaders should advocate for adequate staffing levels, workload management, and institutional accountability to ensure a safer work environment. By integrating these evidence‐based strategies, nursing management can foster a culture of safety, support, and inclusion, ultimately improving workplace conditions, staff well‐being, and patient outcomes.

## 5. Conclusions

This review underscores the pivotal role of cultural factors in influencing the prevalence and dynamics of WPV in healthcare. The evidence presented indicates that WPV is not merely an occupational hazard, but a complex, socioculturally embedded phenomenon requiring tailored interventions at institutional, policy, and sociocultural levels. Ethnic and linguistic disparities, gender norms, and hierarchical structures are significant contributors to WPV, highlighting the need for targeted strategies to address these underlying determinants. Recognizing and addressing the cultural dimensions of WPV will enable the healthcare sector to implement sustainable and inclusive solutions that enhance both worker well‐being and overall patient care quality.

## Disclosure

All authors read and approved the final manuscript. The funders had no role in the design of the study, in the collection, analyses, or interpretation of data, in the writing of the manuscript, or in the decision to publish the results.

## Conflicts of Interest

The authors declare no conflicts of interest.

## Author Contributions

Sara Morales Palomares: writing–original draft preparation, conceptualization, methodology, and reviewing and editing; Flavio Gheri: writing–original draft preparation, conceptualization, and methodology; Nicola Ramacciati: conceptualization, methodology, reviewing and editing, supervision, and project administration.

## Funding

This research did not receive any specific grant from funding agencies in the public, commercial, or not‐for‐profit sectors. Open access publishing was facilitated by Universita della Calabria, as part of the Wiley—CRUI‐CARE agreement.

## Supporting Information

Supporting Table S1. Search strategy. This table reports the full electronic search strategies used for PubMed, CINAHL and Scopus. The supporting information is cited in the text as supporting Table S1 (see Methods, Section 2.4 Search Strategy).

## Supporting information


**Supporting Information** Additional supporting information can be found online in the Supporting Information section.

## Data Availability

All data supporting the reported results have been fully shared and are publicly available in accordance with Wiley Research Data Policies.
