# Supplementary material for: Cultural Dimensions of Workplace Violence in Healthcare: A Narrative Review
Source: J Nurs Manag. 2025 Dec 22;2025:8336854. doi: 10.1155/jonm/8336854 (PMC12721393; doi:10.1155/jonm/8336854)
Supplement: Supplementary file 1 — Supporting Information Additional supporting information can be found online in the Supporting Information section. [file JONM-2025-8336854-s001.docx]

**Supplementary File**

**Table S1. Search strategy**

| **DB** | **STRINGA** | **RESULTS** | **DATA** |
| --- | --- | --- | --- |
| PubMed | (((cultural or culture) and (factor* or caratheristic* or variable*)) AND ("workplace violence" OR "occupational violence")) | 119 | 20/01/2025 |
| Cinahl | ((culture OR cultural) AND ((workplace violence) OR (occupational violence)) | 638 | 20/0172025 |
| Scopus | (culture OR cultural ) AND ( " workplace violence " OR " occupational violence " ) | 374 | 20/01/2025 |
| Total articles found | | 1131 | |
